# Retraction: LncRNA DQ786243 contributes to proliferation and metastasis of colorectal cancer both *in vitro* and *in vivo*

**DOI:** 10.1042/BSR-2016-0048_RET

**Published:** 2024-02-28

**Keywords:** colorectal cancer (CRC), Invasion, long non-coding ribonucleic acid (LncRA), Migration

This Retraction follows an Expression of Concern relating to this article previously published by Portland Press.

This article is being retracted from *Bioscience Reports* at the request of the Editor-in-Chief and the Editorial Board following receipt of a notification from a reader, alerting the Editorial Board to duplications in Figure 3A and 4A. Readers have also identified duplicated images in this article with articles published elsewhere including:

- Figure 2C, SW620 B-actin, with Figure 4E, WM266-4 B-actin, from Du et al. 2017 (doi: 10.1186/s40659-017-0141-8)
- Figure 2C, HT29 B-actin, with Figure 4E, A2058 B-actin, from Du et al. 2017 (doi: 10.1186/s40659-017-0141-8)
- Figure 2C, HT29 Bcl-2, with Figure 7C, HEC-1A AKT, from Huang and Hu 2018 (doi: 10.1042/bsr20171546)
- Figure 4A with Figure 6E from Chang et al. 2017 (doi: 10.1186/s12885-017-3216-6)
- Figure 4B, Blank HT29 and si-DQ786243, with Figure 4A from Wang et al. 2016 (doi: 10.1042/bsr20160165)
- Figure 4B, Blank HT29 and si-DQ786243, with Figure 2F from Chang et al. 2017 (doi: 10.1186/s12885-017-3216-6)

The authors have been contacted with regards to the duplications identified and have agreed to the retraction.

